# Bi-allelic missense disease-causing variants in RPL3L associate neonatal dilated cardiomyopathy with muscle-specific ribosome biogenesis

**DOI:** 10.1007/s00439-020-02188-6

**Published:** 2020-06-08

**Authors:** Mythily Ganapathi, Loukas Argyriou, Francisco Martínez-Azorín, Susanne Morlot, Gökhan Yigit, Teresa M. Lee, Bernd Auber, Alexander von Gise, Donald S. Petrey, Holger Thiele, Lukas Cyganek, María Sabater-Molina, Priyanka Ahimaz, Juan Cabezas-Herrera, Moisés Sorlí-García, Arne Zibat, Markus D. Siegelin, Peter Burfeind, Christie M. Buchovecky, Gerd Hasenfuss, Barry Honig, Yun Li, Alejandro D. Iglesias, Bernd Wollnik

**Affiliations:** 1 Personalized Genomic Medicine, Department of Pathology and Cell Biology, Columbia University Irving Medical Center, New York, 10032 USA; 2 Institute of Human Genetics, University Medical Center Göttingen, Heinrich-Düker-Weg 12, 37073 Göttingen, Germany; 3 Grupo de Enfermedades Raras, Mitocondriales y Neuromusculares (ERMN), Instituto de Investigación Hospital 12 de Octubre (i+12), 28041 Madrid, Spain; 4 Institute of Human Genetics, Hannover Medical School, Carl-Neuberg-Str. 1, 30625 Hannover, Germany; 5 Department of Pediatrics, Columbia University Irving Medical Center, Columbia, 10032 USA; 6 Department of Pediatric Cardiology and Critical Care, Hannover Medical School, Carl-Neuberg-Str. 1, 30625 Hannover, Germany; 7 Department of Systems Biology, Columbia University Irving Medical Center, 1130 Nicholas Ave, Columbia, 10032 USA; 8 Cologne Center for Genomics, University of Cologne, Weyertal 115b, 50931 Cologne, Germany; 9 Clinic for Cardiology and Pneumology, University Medical Center Göttingen, German Center for Cardiovascular Research (DZHK), Partner Site Göttingen, Robert-Koch-Str. 40, 37075 Göttingen, Germany; 10 Department of Cardiology, Hospital Clínico Universitario Virgen de la, Arrixaca, IMIB-Arrixaca, Murcia, Spain; 11 Molecular Therapy and Biomarkers Research Group, Hospital Clínico Universitario Virgen de La Arrixaca, IMIB-Arrixaca, Murcia, Spain; 12 Department of Pediatric Cardiology, Hospital Clínico Universitario Virgen de La Arrixaca, 30120 Murcia, Spain; 13 Department of Pathology and Cell Biology, Columbia University Irving Medical Center, New York, 10032 USA; 14 Department of Systems Biology, Biochemistry and Molecular Biophysics, Medicine, Zuckerman Institute, Columbia University Irving Medical Center, 1130 Nicholas Ave, New York, 10032 USA; 15 Cluster of Excellence Multiscale Bioimaging from: Molecular Machines to Networks of Excitable Cells (MBExC), University of Göttingen, Göttingen, Germany

## Abstract

Dilated cardiomyopathy (DCM) belongs to the most frequent forms of cardiomyopathy mainly characterized by cardiac dilatation and reduced systolic function. Although most cases of DCM are classified as sporadic, 20–30% of cases show a heritable pattern. Familial forms of DCM are genetically heterogeneous, and mutations in several genes have been identified that most commonly play a role in cytoskeleton and sarcomere-associated processes. Still, a large number of familial cases remain unsolved. Here, we report five individuals from three independent families who presented with severe dilated cardiomyopathy during the neonatal period. Using whole-exome sequencing (WES), we identified causative, compound heterozygous missense variants in *RPL3L* (ribosomal protein L3-like) in all the affected individuals. The identified variants co-segregated with the disease in each of the three families and were absent or very rare in the human population, in line with an autosomal recessive inheritance pattern. They are located within the conserved RPL3 domain of the protein and were classified as deleterious by several *in silico* prediction software applications. *RPL3L* is one of the four non-canonical riboprotein genes and it encodes the 60S ribosomal protein L3-like protein that is highly expressed only in cardiac and skeletal muscle. Three-dimensional homology modeling and *in silico* analysis of the affected residues in RPL3L indicate that the identified changes specifically alter the interaction of RPL3L with the RNA components of the 60S ribosomal subunit and thus destabilize its binding to the 60S subunit. In conclusion, we report that bi-allelic pathogenic variants in *RPL3L* are causative of an early-onset, severe neonatal form of dilated cardiomyopathy, and we show for the first time that cytoplasmic ribosomal proteins are involved in the pathogenesis of non-syndromic cardiomyopathies.

**Electronic supplementary material:**

The online version of this article (10.1007/s00439-020-02188-6) contains supplementary material, which is available to authorized users.

## Introduction

Pediatric cardiomyopathies are inherited forms of structural heart diseases. They occur with an incidence of 1–2 in 100,000 individuals and include common presentations such as hypertrophic (HCM) and dilated cardiomyopathy (DCM) as well as rare, infrequent forms such as restrictive (RCM), noncompaction (NCM), mixed and arrhythmogenic right ventricular cardiomyopathies (Lee et al. 2017; Vasilescu et al. 2018). Besides exogenous factors like infection and toxins, pediatric cardiomyopathies can result from germline mutations. Pathogenic variants in a large set of genes have been associated with these conditions, but the yield of genetic testing still remains low, especially for non-syndromic cases (Vasilescu et al. 2018).

Pediatric DCM is a genetically heterogeneous disorder. It can be inherited in different modes including autosomal dominant, autosomal recessive, X-linked and mitochondrial inheritance. This complicates genetic testing and variant interpretation, especially as variants in the same gene can cause different cardiomyopathy-related phenotypes. Mutations in genes encoding for components of the sarcomere, the Z-disc and the desmosome have been identified in DCM as well as pathogenic variants in genes coding for components of the nuclear envelope (Taylor et al. 2007; Bates et al. 2012; Kindel et al. 2012; Towbin 2014). Additionally, DCM can also occur as part of a congenital myopathy as observed, e.g., in patients with Duchenne muscular dystrophy, and mutations in underlying genes can affect both skeletal and heart muscle tissues (Spurney 2011; Barp et al. 2015). Most of the known genetic causes of DCM are autosomal dominantly inherited, though the implementation of next-generation sequencing (NGS)-based approaches has led to the identification of novel genes associated with autosomal recessive DCM, mainly in severe childhood-onset cardiomyopathies (i.e., *LEMD2, ACADVL, CAP2, TAF1A*) (Long et al. 2017; Abdelfatah et al. 2019; Aspit et al. 2019; Carlus et al. 2019; Reza et al. 2019).

Still, the genetic background of severe childhood-onset DCM is poorly understood and largely underdiagnosed. Currently, genetic testing in individuals with DCM who have a positive family history of cardiomyopathy identifies causative mutations in only approximately 25% (Murphy et al. 2016). Therefore, the identification of novel DCM-associated genes and mutation signatures not only impacts on genetic testing and on counseling, but also offers the opportunity to develop novel, disease-specific therapies for structural heart diseases based on new pathophysiological insights obtained by the analysis of the functional role of these genetic factors.

Here, we present bi-allelic pathogenic variants in *RPL3L* in five affected children from three independent families originating from Germany, Colombia, and Spain. All affected children were born to healthy parents and presented with a severe form of early-onset DCM leading to neonatal heart failure. We used a GeneMatcher-based approach (Sobreira et al. 2015) to connect the three centers at the Columbia University Irving Medical Center (New York, USA), the University Medical Center Göttingen (Göttingen, Germany) and the Instituto de Investigación Hospital 12 de Octubre (i + 12) (Madrid, Spain), in which clinical examination of patients and/or genetic analyses took place. In a whole-exome sequencing approach, we were able to show that all five individuals carry compound heterozygous missense variants in *RPL3L* encoding a skeletal and heart muscle-specific component of the 60S ribosomal subunit. We confirmed all identified variants in *RPL3L* by Sanger sequencing and verified their co-segregation with the disease in the respective families. All variants affect highly conserved residues, and three-dimensional homology modeling as well as *in silico* analysis of the affected residues in RPL3L indicate that the changes specifically alter the interaction of RPL3L with the 60S ribosomal subunit and thus destabilize its binding to the 60S subunit.

## Materials and methods

### Subjects

All subjects or their legal representatives gave written informed consent for the molecular genetic analyses and for publication of the results. This study was performed according to the Declaration of Helsinki protocol and approved by the local institutional review boards (Columbia University Irving Medical Center, USA; University Medical Center Göttingen, Germany; Instituto de Investigación Hospital 12 de Octubre (i + 12), Spain). DNA from participating family members was extracted from peripheral blood lymphocytes by standard extraction procedures.

### Whole-exome sequencing

**Fig. 1:**
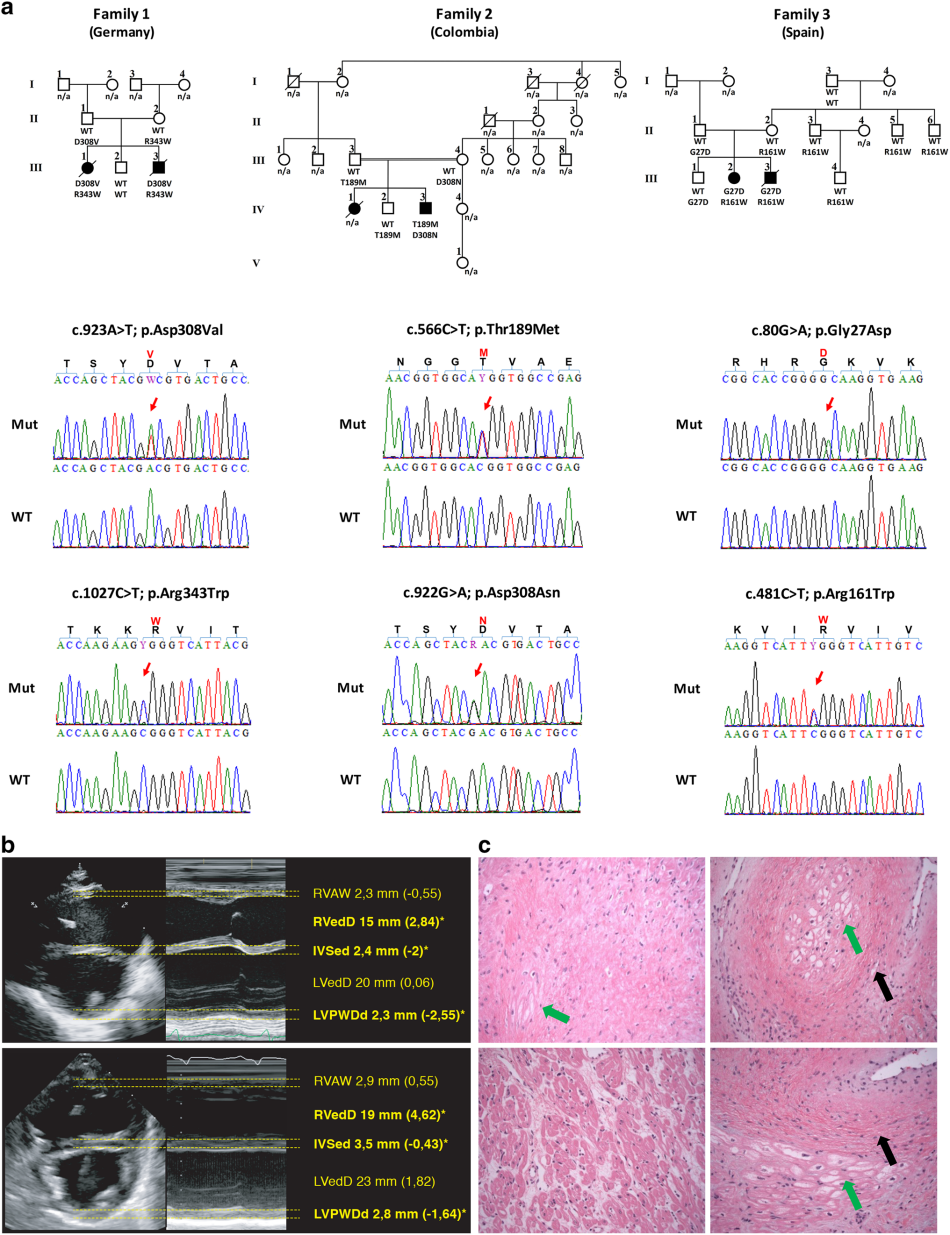
Pedigrees, genetic and clinical characterization of three families with DCM carrying bi-allelic variants in *RPL3L*. **a** Pedigrees of families 1–3, genotypes and electropherograms of the identified *RPL3L* variants. All affected siblings carry compound heterozygous variants in *RPL3L*, while all parents were heterozygous carriers of one of the identified variants. Non-affected siblings were either homozygous for the wild-type allele (individual III.2, family 1) or heterozygous carrier of only one identified *RPL3L* variant (individual IV.2, family 2; individual III.1, family 3). n/a, DNA sample not available. **b** Echocardiogram in parasternal short-axis view of individuals III.1 (upper panel) and III.3 (lower panel) of family 1 including measurements and z-scores. Note end-diastolic dilation of the right ventricle and muscular hypotrophy of the septal and posterior wall. Asterisk and bold letter indicate pathological values. RVAW, right ventricular anterior wall. *RVedD* right ventricular end-diastolic diameter. *IVSed* intraventricular septum end-diastolic. *LVedD* left ventricular end-diastolic dimension. *LVPWDd* left ventricular posterior wall dimension diastole. **c** Pathological evaluation of the explant heart tissue of individual IV.3 (family 2). H & E staining of the myocardium revealed myocytolysis (green arrows) and fibrotic regions (black arrows)

In family 1, WES of both affected children, their non-affected sibling and their parents was carried out using the IDT xGen Exome Research Panel v1.0 enrichment kit (Integrated DNA Technologies) on an Illumina NextSeq500 sequencer (Fig. 1a, left panel). WES data analysis and filtering of variants were carried out using the exome analysis pipeline ‘Varbank 2′ of the Cologne Center for Genomics (CCG, University of Cologne, Germany).

In family 2, trio-based WES was performed at the Laboratory of Personalized Genomic Medicine at Columbia University Irving Medical Center on DNA obtained from peripheral blood lymphocytes of individuals IV.3, III.3 and III.4 (Fig. 1a, middle panel). Exonic and adjacent intronic sequences were enriched from genomic DNA using the Agilent SureSelectXT Human All Exon v5 + UTRs capture kit (Agilent Technologies) according to the manufacturer’s protocol. Paired-end sequencing was performed on the Illumina HiSeq2500 platform. The sequence data were aligned to GRCh37/hg19 and variants were annotated using the Nextgene (version 2.3; SoftGenetics, LLC) software. Variant filtering and annotation were performed using an in-house developed pipeline and reviewed as part of the clinical workflow for constitutional clinical exome sequencing in the laboratory of Personalized Genomic Medicine at Columbia University Irving Medical Center.

In family 3, WES was performed on genomic DNA obtained from patients III.2 and III.3 following a standard protocol (Fig. 1a, right panel). Enrichment was carried out using the Agilent SureSelect Human All Exon V4 enrichment kit (Agilent Technologies), the captured library was sequenced on an Illumina HiSeq2000 platform, and the reads were aligned against the human reference genome (GRCh37/hg19, UCSC) to obtain candidate variants.

### Variant confirmation and Sanger sequencing

Variant confirmations were performed using standard methods for PCR amplification and Sanger sequencing. Primer sequences are available on request. The coding sequence of the respective exons was analyzed and variants were confirmed by a second PCR on an independent DNA sample.

### Prediction programs

*In silico* prediction of the variant effect for all missense variants was evaluated using SIFT, PolyPhen-2, MutationTaster, Combined Annotation Dependent Depletion (CADD), Mendelian Clinically Applicable Pathogenicity (M-CAP), Missense badness, PolyPhen-2, and Constraint (MPC), Missense Variant Pathogenicity2 prediction (MVP2) and PrimateAI (Jagadeesh et al. 2016; Samocha et al. 2017; Qi et al. 2018; Sundaram et al. 2018).

### Three-dimensional homology modeling of human RPL3L

A model of RPL3L was constructed using the structure of RPL3 in the human ribosome (PDB code 3j3b, chain B) (Anger et al. 2013). An alignment of RPL3L to RPL3 was generated with the program bl2seq from the BLAST suite of sequence alignment programs. The model was constructed using the program NEST. To visualize RPL3L in the context of the ribosome, the model of RPL3L was superposed on to the structure of RPL3 using rigid body superposition and the 60S RNA was added from the PDB structure 3j3f, chain 5.

### Databases and web resources

The following databases and web resources were used for this study:

CADD, https://cadd.gs.washington.edu/snv

Clustal Omega, https://www.ebi.ac.uk/Tools/msa/clustalo/

Ensembl, https://www.ensembl.org/index.html

gnomAD browser, https://gnomad.broadinstitute.org/

M-CAP, https://bejerano.stanford.edu/mcap/

MutationTaster, https://www.mutationtaster.org

OMIM, https://www.ncbi.nlm.nih.gov/omim

PolyPhen-2, https://genetics.bwh.harvard.edu/pph2/

SIFT, https://sift.bii.a-star.edu.sg/

SMART, https://smart.embl-heidelberg.de/

UCSC browser, https://genome.ucsc.edu/

UniProt, https://www.uniprot.org/

Varbank2, https://varbank.ccg.uni-koeln.de/varbank2

## Results

### Case reports

Family 1 is a non-consanguineous family of German origin (Fig. 1a, left panel; Table 1). Individual III.1 was the first child born after an uneventful pregnancy. During pregnancy, ultrasound revealed a small patent foramen ovale (PFO). Perinatal monitoring revealed a DCM at the first day of life. Echocardiography showed dilated right ventricle (RV) and pulmonary arterial hypertension (Fig. 1b, upper panel). DCM progressed rapidly after birth leading to dilated left ventricle (LV), an ejection fraction (EF) of 28–32% and cardiac decompensation at the age of 12 days (Fig. 1b, upper panel). Hypotonia was not observed. She died at the age of 21 days. Myocardial pathology assessment showed degenerated myocytes with perinuclear vacuolization, hypertrophic muscle fibers and diffuse interstitial fibrosis. Her brother (individual III.3, Fig. 1a, left panel) was born after normal gestation and uneventful pregnancy. He was diagnosed with DCM and tricuspid insufficiency on the sixth day of life. DCM also progressed rapidly, resulting in massively dilated right atrium (RA) and RV. Left atrium (LA) was slightly dilated, LV was normal (Fig. 1b, lower panel). EF was calculated at 30–36% at the age of 14 days. He showed no signs of hypotonia and died on the 15th day of life due to acute cardiac decompensation. Autopsy confirmed DCM and excluded myocardial infections.

**Table 1:** Summary of genetic data and clinical features of affected individuals

Family	Family 1		Family 2		Family 3	
Pedigree ID	III.1	III.3	IV.1	IV.3	III.2	III.3
Gender	Female	Male	Female	Male	Female	Male
Geographic origin	Germany	Germany	Colombia	Colombia	Spain	Spain
RPL3L variant	c.923A > T (p.Asp308Val) and c.1027C > T (p.Arg343Trp)	c.923A > T (p.Asp308Val) and c.1027C > T (p.Arg343Trp)	N/A	c.566C > T (p.Thr189Met) and c.922G > A (p.Asp308Asn)	c.80G > A; p.Gly27Asp and c.481C > T; p.Arg161Trp	c.80G > A; p.Gly27Asp and c.481C > T; p.Arg161Trp
Dilated cardiomyopathy (age at diagnosis)	+ (1st d.o.l.)	+ (6th d.o.l.)	+ (2 months)	+ (2 ½ months)	+ (1 ½ months)	+ (12th d.o.l.)
Pulmonary arterial hypertension	+	–	N/A	–	+	+
Low ejection fraction	+ (28–32%)	+ (30–36%)	N/A	+ (6%)	+ (30%)	+ (25%)
Cardiac decompensation	+ (12th d.o.l.)	+ (15th d.o.l.)	+ (2 months)	+ (2 ½ months)	+ (1 ½ months)	+ (12th d.o.l.)
Heart valve disease	–	+ (TI)	N/A	+ (TR,MR)	+ (MR)	+ (MR,TR)
Electrocardiography findings	–	–	N/A	RV conduction delay, ST and T abnormalities	ST and T abnormalities	ST and T abnormalities
Additional findings	PFO	–	N/A	lymphoproliferative disorder (post transplantation)	–	VSD (muscular)
Cardiac muscle biopsy	+	–	–	+	–	–
Myocytolysis	+	N/A	N/A	+	N/A	N/A
Interstitial fibrosis	+	N/A	N/A	+	N/A	N/A
Hypertrophic muscle fibers	+	N/A	N/A	+	N/A	N/A
Heart transplantation (age)	–	–	–	+ (5 months)	+ (5 months)	–
Death (age)	+ (21st d.o.l.)	+ (15th d.o.l.)	+ (2 months)	–	–	+ (30th d.o.l.)

*N/A* not available, *d.o.l*. day of life, *TI* tricuspidal insufficiency, *TR* tricuspidal regurgitation, *MR* mitral regurgitation, *PFO* patent foramen ovale, *VSD* ventricular septal defect

Family 2 originated from Colombia and parents were second-degree cousins (Fig. 1a, middle panel; Table 1). Individual IV.3 is the third born child of healthy parents. He was first seen at 2 ½ months of age and presented with non-bilious, non-bloody vomiting and tachypnea. Echocardiogram showed severely decreased biventricular systolic function (EF 6%), moderate LV dilation (LV end-diastolic diameter 3.7 cm; *z*-score 6.9) with mild LA dilation, and mild tricuspid and mitral valve regurgitation. Chest X-ray revealed cardiomegaly with diffusely increased vascularity and a small right pleural effusion. Electrocardiogram showed right axis deviation with RV conduction delay, prominent LV forces for age and nonspecific ST and T wave abnormalities. At 5 months of age, he underwent ABO-incompatible heart transplant. Pathology evaluation of the explant heart showed moderate myocyte hypertrophy, interstitial fibrosis, and multi-focal subendocardial myocytolysis with evidence of chronic LV ischemia (Fig. 1c). The LV endocardium was more thickened and fibrotic than the RV. Metabolic testing, chromosomal microarray, and clinical cardiomyopathy gene panel were unremarkable except for the presence of long contiguous regions of homozygosity in multiple chromosomes, consistent with known consanguinity of the parents. He is currently 9 years of age, and his post-transplant course has been complicated by lymphoproliferative disorder confirmed on ileal biopsy, eosinophilic esophagitis, pancreatitis, left hydronephrosis, and a chronic anemia. We did not observe any signs of hypotonia or muscle issues, and measurements of creatine kinase (CK) level at the ages of 2 months, 5, 6 and 7 years were essentially normal. His sister (individual IV.1, Fig. 1a, middle panel), the first-born child, was born after an uneventful pregnancy. At 2 months of age, she presented with fatigue, vomiting, and lethargy. Heart failure was detected and she died 6 days later. Her clinical care was entirely in Colombia and medical records are not available. The unaffected brother (individual IV.2; Fig. 1a, middle panel) is a 10-year-old boy, who underwent cardiac screening around 2 years of age. He is currently clinically unremarkable.

Family 3 is a non-consanguineous family of Spanish origin (Fig. 1a, right panel; Table 1). The affected individual III.2 is the second-born child of healthy parents. She was born after an uneventful pregnancy and was admitted due to cardiogenic shock on her 48th day of life. She was diagnosed with DCM with severe ventricular dysfunction and pulmonary blood pressure at 50% of the systemic pressure. Orthotopic heart transplantation was performed at 5 months of age. Currently, she is 10 years old. Her brother, the third-born child, was admitted with the diagnosis of DCM and secondary heart failure on 12th day of life (individual III.3, Fig. 1a, right panel). His clinical state suffered rapid deterioration due to cardiogenic shock, which normalized after respiratory and hemodynamic stabilization. Subsequently, he suffered two cardiac arrests and died on the 14th day of life.

As the pedigrees of all three families suggested a genetic factor causative of the DCM, we performed NGS-based analyses of affected and healthy family members to determine possible genetic factors involved in the pathogenesis of DCM in these families.

### WES analysis

In family 1, we performed WES on DNA extracted from blood lymphocytes of both affected children, their non-affected sibling and their parents. WES data analysis and filtering of variants were carried out using the exome analysis pipeline ‘Varbank 2′ (Cologne Center for Genomics University of Cologne, Germany). We obtained a mean coverage of 93–106 reads, and 98.2%–99.0% of targets were covered more than 10x. After exclusion of de novo, homozygous or compound heterozygous variants in known genes associated with DCM, WES data were filtered for variants with a coverage of more than 6 reads, a minimum quality score of 10, an allele frequency ≥ 25%, a minor allele frequency (MAF) < 0.5% in the gnomAD database (Karczewski et al. 2019), and no annotation in the in-house WES datasets of the CCG. We did not detect any homozygous or de novo variants that were shared in the affected individuals and absent in the healthy sibling. Analysis for compound heterozygous variants revealed putative causative variants in only one gene, *RPL3L*. Both affected siblings carried compound heterozygous variants, c.923A > T and c.1027C > T, in *RPL3L* inherited either from their father (c.923A > T) or mother (c.1027C > T), whereas the non-affected sibling carried the wild-type sequence on both alleles (Fig. 1a, left panel).

In family 2, trio-based WES of the affected individual IV.3 and both parents was performed at the Laboratory of Personalized Genomic Medicine at Columbia University Irving Medical Center (Fig. 1a, middle panel). Identified variants were assessed for clinical phenotypic match and American College of Medical Genetics and Genomics (ACMG) guidelines for the interpretation of sequence variants (Richards et al. 2015). WES data were filtered for de novo, homozygous or compound heterozygous variants with allele frequencies of less than 1% in the databases of the 1000 Genomes project and the Exome Variant Server (EVS; NHLBI Exome Sequencing Project). We excluded pathogenic or likely pathogenic variants in any of the known genes associated with cardiomyopathy and subsequently analyzed the WES data for novel putative causative variants. This analysis revealed compound heterozygous variants in *RPL3L* in individual IV.3. The index patient carried the paternally inherited c.566C > T variant and the maternally inherited c.922G > A variant in *RPL3L*. We confirmed these variants by Sanger sequencing and co-segregation analysis revealed heterozygous carrier status for one variant (c.566C > T) in the healthy sibling IV.2 (Fig. 1a, middle panel).

In family 3, we performed WES on genomic DNA obtained from patients III.2 and III.3 (Fig. 1a, right panel) and we applied the following criteria for filtering of the WES data: a minor allele frequency (MAF) < 1% in the gnomAD database (v2.1.1), a predicted impact on protein function (including nonsense, splice-site, coding indel, or missense variants), and consistent with a recessive (homozygous or compound heterozygous variants) or dominant model of pathogenesis (heterozygous variants with a MAF < 0.001%). Using this analytic pipeline, we were able to identify two heterozygous single nucleotide variations, c.80G > A and c.481C > T, in the *RPL3L* gene. Sanger sequencing of patient and parental DNA confirmed compound heterozygosity of these variants in both affected individuals as well as heterozygous carrier status of each parent for one of the identified variants (Fig. 1a, right panel). The healthy sibling (individual III.1, Fig. 1a, right panel) was a heterozygous carrier for the c.80G > A variant in *RPL3L*.

**Fig. 2:**
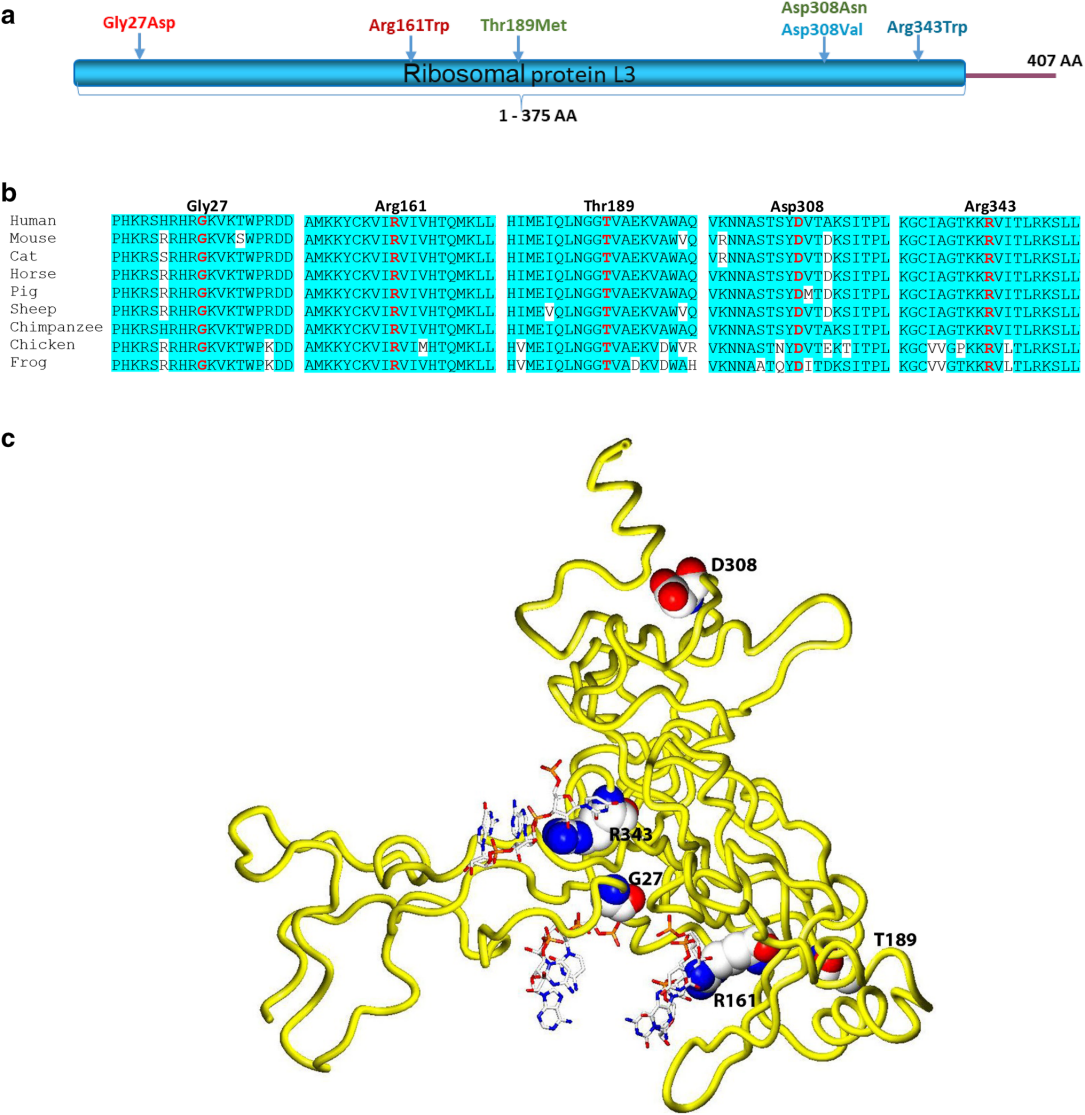
Molecular characterization of the identified RPL3L variants. **a** Schematic RPL3L protein structure and the localization of the identified variants. All missense variants are located within the conserved RPL3 domain of the protein. **b** Amino acid sequence alignment of RPL3L residues that are altered in the affected individuals including surrounding residues across different species. **c** Three-dimensional homology model of the RPL3L protein. RPL3L is shown in worm representation (yellow). Affected amino acid residues are labeled in black, shown in sphere representation and colored according to atom type (blue = nitrogen, white = carbon, *red* = oxygen). Portions of the 60S RNA in proximity to altered residues (R343, R161 and G27) are shown in ball-and-stick representation and colored according to atom type

In all five affected individuals presenting with DCM, we identified compound heterozygous variants in *RPL3L*. The identified variants co-segregated with the disease in each of the three families. On protein level, all variants are located within the *ribosomal protein L3* domain of RPL3L and are predicted to lead to the substitution of phylogenetically highly conserved amino acids in RPL3L (Fig. 2a,b). Interestingly, two variants, c.923A > T identified in family 1 and c.922G > A identified in family 2, affect the same amino acid residue p.Asp308 substituting it either by valine (family 1) or asparagine (family 2), which provides additional genetic evidence for the causality of both variants. All six variants are very rare in the general human population with minor allele frequencies (MAFs) ranging from 0 to 4.25*10^–5^, in line with an autosomal recessive inheritance pattern (Table 2). *In silico* prediction using different prediction tools leads to consistent variant classification of all six variants as damaging (SIFT), probably/possibly damaging (PolyPhen-2), disease causing (MutationTaster), and a Combined Annotation Dependent Depletion (CADD; v1.4) score ranging from 23.6 to 24.9, respectively, indicating deleteriousness of these variants (Table 2). Similarly, classification of these variants using the M-CAP (Mendelian Clinically Applicable Pathogenicity) score, the MVP2 (Missense Variant Pathogenicity2) prediction, the MPC (Missense badness, PolyPhen-2, and Constraint) score as well as the PrimateAI prediction score confirmed the deleterious effect of these missense variants (Table S1, S2).

**Table 2:** *In silico* prediction and population allele frequencies of *RPL3L* variants reported in this study

Family	Genomic location (hg19)	HGVS cDNA (NM_005061.3)	HGVS protein (NP_005052.1)	Allele frequency in the gnomAD database^a^	Prediction scores			
					SIFT^b^	PolyPhen-2^c^	MutationTaster	CADD
1	Chr16:1,996,654	c.923A > T	p.(Asp308Val)	0	D (0)	PD (0.998)	Disease causing	23.7
	Chr16:1,995,856	c.1027C > T	p.(Arg343Trp)	1 in 251,232	D (0)	PD (0.997)	Disease causing	23.6
2	Chr16:1,997,317	c.566C > T	p.(Thr189Met)	6 in 248642^d^	D (0)	PD (0.912)	Disease causing	24.0
	Chr16:1,996,655	c.922G > A	p.(Asp308Asn)	0	D (0)	PD (0.993)	Disease causing	24.1
3	Chr16:2,000,865	c.481C > T	p.(Arg161Trp)	12 in 282644^d^	D (0)	PoD (0.905)	Disease causing	24.9
	Chr16:2,004,073	c.80G > A	p.(Gly27Asp)	2 in 229842^d^	D (0)	PD (0.997)	Disease causing	24.4

^a^Accessed in April 2020, gnomAD v2.1.1

^b^Score 1–0, *D* deleterious

^c^HumVar prediction, Score 0–1, *PD* probably damaging, *PoD* possibly damaging

^d^Only in heterozygous state

### *In silico* structural analysis of the identified RPL3L variants

RPL3L variants reported in our affected individuals with DCM are spread throughout the protein (Fig. 2a). To analyze the structural and functional impact of these RPL3L missense variants, we performed three-dimensional homology modeling of human RPL3L. RPL3L is the paralog of RPL3*,* and both proteins share 78% identity at the amino acid level. RPL3 is a ubiquitously expressed 60S ribosomal subunit, which forms a crucial component of the ribosomal peptidyltransferase center and fulfills an essential coordinating function as a “gatekeeper” to the A site of ribosomes (Meskauskas and Dinman 2007). Its structure as part of the 60S ribosomal subunit has been well established (Anger et al. 2013). As the amino acid position of all six6 missense variants which we identified in RPL3L were conserved in both paralogs, we constructed a homology-based structural model of RPL3L using the structure of RPL3 in the human ribosome to gain further insights into the pathogenic effects of the identified RPL3L missense variants (Altschul et al. 1997; Petrey et al. 2003). Three mutated residues were located in regions directly contributing to RNA binding. *In silico* analysis showed that the basic residues p.Arg161 and p.Arg343 of RPL3L form salt bridges with the RNA phosphate backbone, stabilizing RPL3L binding to the RNA. Mutation of these residues to tryptophan, as identified in our patients, leads to the loss of these basic, positively charged arginine residues, destabilizing the binding to the RNA and thereby weakening the binding of RPL3L to the 60S subunit (Fig. 2c; Fig. S1). Also, the p.Gly27 residue is located in proximity to the ribosomal RNA, and introduction of a negatively charged aspartate at this position, as identified in family 3, generates a negative charge in proximity to the RNA phosphate backbone leading to an electrostatic repulsion that, again, potentially weakens the binding of RPL3L to the ribosomal RNA (Fig. 2c; Fig. S1). Thus, these three *RPL3L* variants are highly likely to weaken binding of RPL3L to the ribosome either by removing a favorable charge–charge interaction between the arginines and the phosphate backbone of the RNA [p.(Arg161Trp), p.(Arg343Trp)] or by introducing a charge–charge repulsion with the negatively charged phosphates [p.(Gly27Asp); Fig. 2c, Fig. S1].

Three variants, p.Thr189Met, p.Asp308Asn and p.Asp308Val, are not located in the tentacle-like structure of RPL3L that interacts with the RNA, but in the large globular domain on the cytoplasmic face of the complex (Fig. 2c). The Asp308Asn and p.Asp308Val substitutions remove an anionic residue, and both are predicted to cause structural perturbation of the region, potentially impairing interactions between RPL3L and other proteins components of the 60S ribosomal subunit. Interestingly, the threonine residue at position 189 of RPL3 has been determined as a phosphorylation site in a large-scale phosphoproteome analysis (Olsen et al. 2010). The exchange of this threonine residue to methionine in RPL3L, as observed in patient IV.3 of family 2, abrogates this potential phosphorylation site and might thereby interfere with RPL3L function. Still, it is unknown whether this phosphorylation also takes place in RPL3L, and additional functional analyses are needed to determine the functional consequences that are associated with the disruption of this potential phosphorylation site. Overall, all reported RLP3L amino acid substitutions affect highly conserved positions and are predicted to perturb the structure of the RPL3L subunit and its binding to other components of the 60S ribosomal subunit; however, further functional studies are needed to address the functional consequences of the identified variants in RPL3L.

## Discussion

In this report, we provide evidence that bi-allelic mutations in *RPL3L* cause a severe dilated cardiomyopathy during the neonatal period. In five affected individuals from three independent families, we identified compound heterozygous missense variants in *RPL3L* and showed by three-dimensional homology modeling that these missense variants destabilize RPL3L binding to the 60S ribosomal subunit.

RPL3L is a paralog to RPL3, a highly conserved, ubiquitously expressed ribosomal protein that is a component of the 60S ribosomal subunit (Brodersen and Nissen 2005). In contrast to RPL3, RPL3L is specifically expressed in skeletal muscle and heart tissue (Van Raay et al. 1996; Gupta and Warner 2014). Expression analysis in these tissues revealed that *RPL3L* mRNA levels are not static, but regulated dynamically in response to external stimuli (Chaillou et al. 2016). In response to hypertrophic stimuli, *Rpl3l* mRNA is dramatically downregulated suggesting a role of Rpl3l as a negative regulator of muscle growth (Chaillou et al. 2016). Additionally, exogenous expression of RPL3L in C2C12 myogenic cells during differentiation leads to its incorporation in ribosomes and impairs myotube growth and fusion (Chaillou et al. 2016). Interestingly, expression of RPL3L and RPL3 are conversely regulated. Downregulation of RPL3L in response to hypertrophic stimulus induces concurrent upregulation of *RPL3* mRNA. This observation is in line with the concept of the “ribosomal code”, which postulates that ribosomal function and specificity can be regulated based on ribosomal protein composition, post-translational modification of ribosomal components, and alternate rRNA forms, which in turn has an influence on subsets of mRNA that are preferentially translated (Mauro and Edelman 2002; Xue and Barna 2012; Sauert et al. 2015). Currently, we can only speculate about the specific mRNAs that are preferentially translated by ribosomes containing RPL3L instead of RPL3, and further experimental studies are needed to determine how expression levels of RPL3L and RPL3 are regulated in skeletal muscle and heart tissue.

To date, no Mendelian disorder has been associated with mutations in RPL3L. Overall, *RPL3L* homozygous loss-of-function variants are not commonly seen in the gnomAD (access date 03/04/2020) and TOPMed (access date 03/04/2020) databases except for one homozygous variant, c.1167 + 1G > A, in intron 9 of *RPL3L*. This *RPL3L* variant has been detected in 347 of 275,594 alleles within the gnomAD database (MAF = 0.001259). Of note, this variant was recently associated with increased risk of atrial fibrillation (Thorolfsdottir et al. 2018). It induces alternative splicing of *RLP3L* pre-mRNA, leading to skipping of coding exon 9 which results in an in-frame deletion of 40 amino acids, p.(Ser350_Met389del), in RPL3L. Analyzing *RPL3L* expression in cardiac RA tissue of two heterozygous carriers of the c.1167 + 1G > A variant, Thorolfsdottir et al. showed that the alternatively spliced *RPL3L* transcript is stable and expressed in approximately equal abundance compared to the full-length *RPL3L* transcript (Thorolfsdottir et al. 2018). They suggested that deletion of these 40 amino acid might disrupt the interaction of RPL3L-containing ribosomes with the endoplasmic reticulum, thereby leading to reduced ribosomal function (Thorolfsdottir et al. 2018).

This is, to the best of our knowledge, the first study to provide evidence of a cytoplasmic ribosomal protein involvement in the pathogenesis of non-syndromic cardiomyopathy. The presented data, along with the common phenotype of severe neonatal DCM with rapid decompensation in all three families, strongly support pathogenicity of the described *RPL3L* variants; still, additional functional studies are needed to analyze the detailed pathomechanisms underlying RPL3L-associacted DCM. Furthermore, confirming the involvement of ribosomal factors in the pathogenesis of DCM possibly reveals a novel disease-associated mechanism, which might lead to the identification of additional genetic factors involved in the pathogenesis of DCM, and, additionally, pave the way for novel therapeutic options and treatment strategies for patients with DCM.

## Electronic supplementary material

Below is the link to the electronic supplementary material.

Supplementary file1 (DOCX 19 kb)

Supplementary file2 (DOCX 1626 kb)
