# Supplementary material for: Bi-allelic missense disease-causing variants in RPL3L associate neonatal dilated cardiomyopathy with muscle-specific ribosome biogenesis
Source: Hum Genet. 2020 Jun 8;139(11):1443–54. doi: 10.1007/s00439-020-02188-6 (PMC7519902; doi:10.1007/s00439-020-02188-6)
Supplement: Supplementary file 1 — Supplementary file1 (DOCX 19 kb) [file 439_2020_2188_MOESM1_ESM.docx]

| *A2ML1* | *AMPD1* | *CACNA1C* | *COA5* | *DOLK* | *FGA* | *GDF2* | *INSR* | *KMT2D* |
| --- | --- | --- | --- | --- | --- | --- | --- | --- |
| *AARS2* | *ANGPTL3* | *CACNA1D* | *COA6* | *DPM3* | *FGF12* | *GFM1* | *IRX3* | *KRAS* |
| *ABCA1* | *ANK2* | *CACNA2D1* | *COL1A1* | *DPP6* | *FHL1* | *GJA1* | *IRX4* | *LAMA2* |
| *ABCB1* | *ANK2* | *CACNB2* | *COL1A2* | *DSC2* | *FHL2* | *GJA5* | *ISL1* | *LAMA4* |
| *ABCC6* | *ANK3* | *CALM1* | *COL3A1* | *DSG2* | *FHOD3* | *GLA* | *ISM2* | *LAMP2* |
| *ABCC9* | *ANKRD1* | *CALM2* | *COL5A1* | *DSP* | *FKBP14* | *GLB1* | *JAG1* | *LCAT* |
| *ABCG1* | *ANO5* | *CALM3* | *COL5A2* | *DTNA* | *FKRP* | *GLIS3* | *JARID2* | *LDB3* |
| *ABCG5* | *APOA1* | *CALR* | *COL7A1* | *DYSF* | *FKTN* | *GNPTAB* | *JPH2* | *LDLR* |
| *ABCG8* | *APOA4* | *CALR3* | *COQ2* | *EEF1A2* | *FLNA* | *GPD1* | *JUP* | *LDLRAP1* |
| *ACAD9* | *APOA5* | *CAP2* | *COX15* | *EFEMP2* | *FLNC* | *GPD1L* | *KANSL1* | *LEFTY2* |
| *ACADVL* | *APOB* | *CASQ2* | *COX6B1* | *EHMT1* | *FOXC1* | *GPIHBP1* | *KAT6B* | *LEMD2* |
| *ACTA1* | *APOC2* | *CASZ1* | *CPT2* | *EIF2AK3* | *FOXD4* | *GREM2* | *KCNA5* | *LEP* |
| *ACTA2* | *APOC3* | *CAV1* | *CREB3L3* | *EIF2AK4* | *FOXE3* | *GSK3B* | *KCND2* | *LIAS* |
| *ACTC1* | *APOE* | *CAV3* | *CREBBP* | *ELAC2* | *FOXF1* | *GUSB* | *KCND3* | *LIPA* |
| *ACTN2* | *ASPH* | *CAVIN1* | *CRELD1* | *ELN* | *FOXH1* | *HADHA* | *KCNE1* | *LIPC* |
| *ACVR1* | *ATP5F1E* | *CAVIN4* | *CRYAB* | *EMD* | *FOXP1* | *HAND1* | *KCNE2* | *LMF1* |
| *ACVR2B* | *ATP7A* | *CBL* | *CSRP3* | *ENG* | *FOXP3* | *HAND2* | *KCNE3* | *LMNA* |
| *ACVRL1* | *ATPAF2* | *CBS* | *CTF1* | *EP300* | *FOXRED1* | *HCN4* | *KCNE5* | *LMOD2* |
| *ADAMTS2* | *B3GAT3* | *CDC42* | *CTNNA1* | *EPG5* | *FXN* | *HFE* | *KCNH2* | *LOX* |
| *ADAMTSL4* | *B4GALT7* | *CDH2* | *CTNNA3* | *EPHB4* | *GAA* | *HNF1A* | *KCNJ11* | *LPA* |
| *AGK* | *BAG3* | *CEL* | *CTNNB1* | *ETFA* | *GATA4* | *HNF1B* | *KCNJ2* | *LPL* |
| *AGL* | *BLK* | *CETP* | *CYP2D6* | *ETFB* | *GATA5* | *HNF4A* | *KCNJ5* | *LRP6* |
| *AGPAT2* | *BMP10* | *CFC1* | *CYP3A4* | *ETFDH* | *GATA6* | *HRAS* | *KCNJ8* | *LRRC10* |
| *AKAP9* | *BMPR1A* | *CH25H* | *CYP3A5* | *EVC* | *GATAD1* | *HSPB8* | *KCNK17* | *LTBP2* |
| *AKT1* | *BMPR1B* | *CHD7* | *DES* | *EYA4* | *GATC* | *IDH2* | *KCNK3* | *LTBP3* |
| *AKT2* | *BMPR2* | *CHRM2* | *DLD* | *FAH* | *GBE1* | *IER3IP1* | *KCNQ1* | *LZTR1* |
| *ALG10B* | *BRAF* | *CHST14* | *DMD* | *FBN1* | *GCK* | *ILK* | *KLF10* | *MAP2K1* |
| *ALMS1* | *BSCL2* | *CIDEC* | *DNAJC19* | *FBN2* | *GCKR* | *INS* | *KLF11* | *MAP2K2* |
| *ALPK3* | *C10orf71* | *CITED2* | *DNM1L* | *FBXO32* | *GDF1* | *INSIG2* | *KLHL24* | *MAP3K8* |
| *MAT2A* | *MTTS2* | *NOS1AP* | *PLOD1* | *RRAD* | *SLC25A3* | *TBC1D4* | *TRIM54* |  |
| *MCTP2* | *MURC* | *NOTCH1* | *PLTP* | *RRAS* | *SLC25A4* | *TBX1* | *TRIM63* |  |
| *MED12* | *MYBPC3* | *NOTCH2* | *PMM2* | *RRAS2* | *SLC25A40* | *TBX20* | *TRPM4* |  |
| *MED13L* | *MYBPHL* | *NOTCH3* | *PNPLA2* | *RYR1* | *SLC2A10* | *TBX3* | *TSFM* |  |
| *MEF2A* | *MYH11* | *NPC1L1* | *PPARA* | *RYR2* | *SLC2A2* | *TBX5* | *TTN* |  |
| *MEF2C* | *MYH6* | *NPHP4* | *PPARG* | *SALL4* | *SLC39A13* | *TCAP* | *TTR* |  |
| *MFAP5* | *MYH7* | *NPPA* | *PPCS* | *SAR1B* | *SLC4A3* | *TDGF1* | *TXNRD2* |  |
| *MIB1* | *MYL2* | *NRAP* | *PPP1CB* | *SCARB1* | *SLCO1B1* | *TECRL* | *UPF3B* |  |
| *MLYCD* | *MYL3* | *NRAS* | *PPP1R13L* | *SCN10A* | *SLMAP* | *TFAP2B* | *VARS2* |  |
| *MRPL3* | *MYL4* | *OBSCN* | *PRDM16* | *SCN1B* | *SLMAP* | *TGFB2* | *VCL* |  |
| *MRPL44* | *MYLIP* | *OPA3* | *PRKAG2* | *SCN2B* | *SMAD1* | *TGFB3* | *VPS13A* |  |
| *MRPS14* | *MYLK* | *PAX4* | *PRKAR1A* | *SCN3B* | *SMAD2* | *TGFBR1* | *WFS1* |  |
| *MRPS22* | *MYLK2* | *PCCA* | *PRKG1* | *SCN4B* | *SMAD3* | *TGFBR2* | *WISP1* |  |
| *MTM1* | *MYO6* | *PCCB* | *PSEN1* | *SCN5A* | *SMAD4* | *TJP1* | *WT1* |  |
| *MTND1* | *MYOM1* | *PCDH15* | *PSEN2* | *SCO1* | *SMAD6* | *TMEM175* | *XK* |  |
| *MTND5* | *MYOT* | *PCSK9* | *PTF1A* | *SCO2* | *SMAD9* | *TMEM43* | *ZBTB17* |  |
| *MTND6* | *MYOZ2* | *PDGFRA* | *PTPN11* | *SDHA* | *SNTA1* | *TMEM43* | *ZDHHC9* |  |
| *MTO1* | *MYPN* | *PDHA1* | *PYGM* | *SEMA3A* | *SOS1* | *TMEM70* | *ZFHX3* |  |
| *MTTD* | *NDUFB11* | *PDLIM3* | *QRSL1* | *SEPN1* | *SOS2* | *TMOD1* | *ZFPM2* |  |
| *MTTG* | *NEBL* | *PDX1* | *RAF1* | *SGCA* | *SPEG* | *TMPO* | *ZHX3* |  |
| *MTTH* | *NEUROD1* | *PERP* | *RANGRF* | *SGCB* | *SPRED1* | *TNNC1* | *ZIC3* |  |
| *MTTI* | *NEUROG3* | *PHKA1* | *RASA1* | *SGCB* | *SPRY1* | *TNNI3* | *ZMPSTE24* |  |
| *MTTK* | *NEXN* | *PITX2* | *RASA2* | *SGCD* | *SREBF2* | *TNNI3K* |  |  |
| *MTTL1* | *NF1* | *PKD2* | *RBCK1* | *SGCD* | *SURF1* | *TNNT2* |  |  |
| *MTTL2* | *NKX2-5* | *PKP2* | *RBM20* | *SGCG* | *SYNE1* | *TOPBP1* |  |  |
| *MTTM* | *NKX2-6* | *PKP4* | *RBM24* | *SHOC2* | *SYNE2* | *TOR1AIP1* |  |  |
| *MTTP* | *NNT* | *PLEKHM2* | *RFX6* | *SKI* | *SYNGAP1* | *TPM1* |  |  |
| *MTTQ* | *NODAL* | *PLIN1* | *RIT1* | *SLC22A5* | *TAB2* | *TRDN* |  |  |
| *MTTS1* | *NONO* | *PLN* | *RMND1* | *SLC22A8* | *TAZ* | *TRIB1* |  |  |
