# Supplementary material for: Bi-allelic missense disease-causing variants in RPL3L associate neonatal dilated cardiomyopathy with muscle-specific ribosome biogenesis
Source: Hum Genet. 2020 Jun 8;139(11):1443–54. doi: 10.1007/s00439-020-02188-6 (PMC7519902; doi:10.1007/s00439-020-02188-6)
Supplement: Supplementary file 2 — Supplementary file2 (DOCX 1626 kb) [file 439_2020_2188_MOESM2_ESM.docx]

**Supplementary Material**

**Fig. S1.** **Homology model of RPL3L binding to the ribosome.**


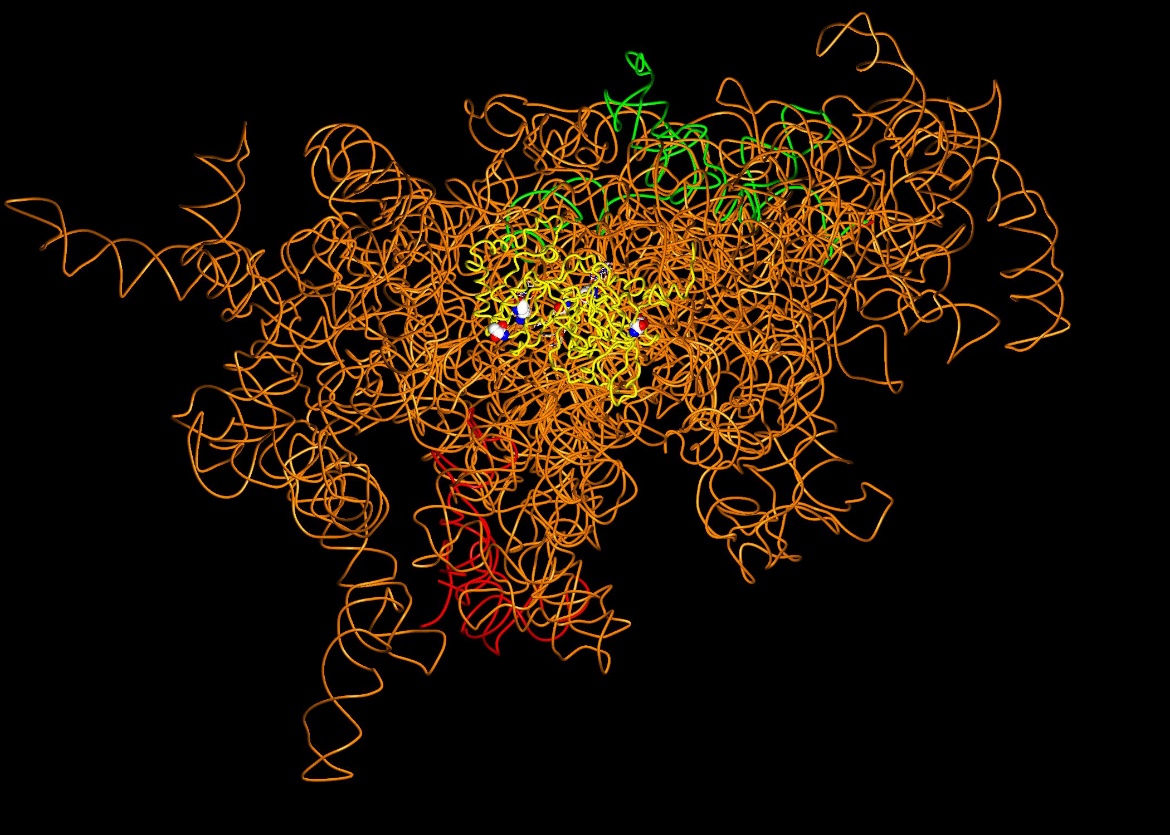


RPL3L and ribosomal RNA are shown in worm representation with RPL3L in yellow, the 28S RNA in orange, the 5S RNA in red, and the 5.8S RNA in green. Residues in RPL3L with significant variants are shown in sphere representation and colored by atom type.

**Table S1.** M-CAP scores and *in silico* prediction of the *RPL3L* variants identified in this study.

| **Family** | **Genomic location (hg19)** | **HGVS cDNA** | **HGVS protein** | **M-CAP scores** | **M-CAP prediction** |
| --- | --- | --- | --- | --- | --- |
|  |  |  |  |  |  |
| **1** | Chr16:1996654 | c.923A>T | p.(Asp308Val) | 0.061 | Possibly Pathogenic |
|  | Chr16:1995856 | c.1027C>T | p.(Arg343Trp) | 0.054 | Possibly Pathogenic |
| **2** | Chr16:1997317 | c.566C>T | p.(Thr189Met) | 0.03 | Possibly Pathogenic |
|  | Chr16:1996655 | c.922G>A | p.(Asp308Asn) | 0.044 | Possibly Pathogenic |
| **3** | Chr16:2000865 | c.481C>T | p.(Arg161Trp) | 0.064 | Possibly Pathogenic |
|  | Chr16:2004073 | c.80G>A | p.(Gly27Asp) | 0.09 | Possibly Pathogenic |

**Table S2.** MPC, MVP2 and PrimateAI prediction scores for the identified RPL3L missense variants.

| **Family** | **Genomic location (hg19)** | **HGVS cDNA** | **HGVS protein** | **MPC** | **MVP2** | **PrimateAI** |
| --- | --- | --- | --- | --- | --- | --- |
|  |  |  |  |  |  |  |
| **1** | Chr16:1996654 | c.923A>T | p.(Asp308Val) | 0.63 | 0.78 | 0.62 |
|  | Chr16:1995856 | c.1027C>T | p.(Arg343Trp) | 0.54 | 0.81 | 0.79 |
| **2** | Chr16:1997317 | c.566C>T | p.(Thr189Met) | 0.52 | 0.25 | 0.57 |
|  | Chr16:1996655 | c.922G>A | p.(Asp308Asn) | 0.43 | 0.65 | 0.67 |
| **3** | Chr16:2000865 | c.481C>T | p.(Arg161Trp) | 0.45 | 0.84 | 0.79 |
|  | Chr16:2004073 | c.80G>A | p.(Gly27Asp) | 0.55 | 0.79 | 0.81 |
